# Can adjuncts to bowel preparation for colonoscopy improve patient experience and result in superior bowel cleanliness? A systematic review and meta-analysis

**DOI:** 10.1177/2050640620953224

**Published:** 2020-08-24

**Authors:** Umair Kamran, Abdullah Abbasi, Imran Tahir, James Hodson, Keith Siau

**Affiliations:** 1 University Hospitals NHS Foundation Trust Birmingham, Birmingham, UK; 2 Department of Gastroenterology, Shrewsbury and Telford NHS Trust, Shrewsbury, UK; 3 Department of Gastroenterology, Worcestershire Acute Hospitals NHS Foundation Trust, Worcester, UK; 4 Medical Statistics, University of Birmingham, Birmingham, UK; 5 Medical and Dental Sciences, University of Birmingham, Birmingham, UK

**Keywords:** Colonoscopy, bowel preparation, patient experience, screening, endoscopy

## Abstract

**Background:**

Bowel preparation for colonoscopy is often poorly tolerated due to poor palatability and adverse effects. This can negatively impact on the patient experience and on the quality of bowel preparation. This systematic review and meta-analysis was carried out to assess whether adjuncts to bowel preparation affected palatability, tolerability and quality of bowel preparation (bowel cleanliness).

**Methods:**

A systematic search strategy was conducted on PubMed, MEDLINE, EMBASE and the Cochrane Database of Systematic Reviews to identify studies evaluating adjunct use for colonoscopic bowel preparation. Studies comparing different regimens and volumes were excluded. Specific outcomes studied included palatability (taste), willingness to repeat bowel preparation, gastrointestinal adverse events and the quality of bowel preparation. Data across studies were pooled using a random-effects model and heterogeneity assessed using *I*^2^-statistics.

**Results:**

Of 467 studies screened, six were included for analysis (all single-blind randomised trials; *n* = 1187 patients). Adjuncts comprised citrus reticulata peel, orange juice, menthol candy drops, simethicone, Coke Zero and sugar-free chewing gum. Overall, adjunct use was associated with improved palatability (mean difference 0.62, 95% confidence interval 0.29–0.96, *p* < 0.001) on a scale of 0–5, acceptability of taste (odds ratio 2.75, 95% confidence interval: 1.52–4.95, *p* < 0.001) and willingness to repeat bowel preparation (odds ratio 2.92, 95% confidence interval: 1.97–4.35, *p* < 0.001). Patients in the adjunct group reported lower rates of bloating (odds ratio 0.48, 95% confidence interval: 0.29–0.77, *p* = 0.003) and vomiting (odds ratio 0.47, 95% confidence interval 0.27–0.81, *p* = 0.007), but no difference in nausea (*p* = 0.10) or abdominal pain (*p* = 0.62). Adjunct use resulted in superior bowel cleanliness (odds ratio 2.52, 95% confidence interval: 1.31–4.85, *p* = 0.006). Heterogeneity varied across outcomes, ranging from 0% (vomiting) to 81% (palatability), without evidence of publication bias. The overall quality of evidence was rated moderate.

**Conclusion:**

In this meta-analysis, the use of adjuncts was associated with better palatability, less vomiting and bloating, willingness to repeat bowel preparation and superior quality of bowel preparation. The addition of adjuncts to bowel preparation may improve outcomes of colonoscopy and the overall patient experience.

## Key summary

### What is already known?

Bowel preparation is often poorly tolerated due to its taste and side effects which can result in inadequate colonoscopic examination and poor patient experience.

### What is new here?

The use of adjuncts with bowel preparation was associated with improved patient experience and better quality of bowel cleanliness.

## Introduction

Colonoscopy is the gold standard modality for investigating the lower gastrointestinal tract with approximately one million procedures performed in the United Kingdom each year.^1^ Despite the intrusive nature of the procedure, many patients perceive the consumption of pre-procedural bowel preparation to be the most burdensome aspect of colonoscopy,^2^,^3^ with poor palatability (taste) being a major challenge.^4^ Issues with palatability can result in nausea, failure to complete the prescribed regimen and a negative experience prior to colonoscopy. In turn, this may impact on mucosal views, procedural completion and missed lesions during colonoscopy. As such, improving the palatability of bowel preparation may improve patient acceptance of colonoscopy and other patient-centred outcomes.

Currently, most bowel preparation regimens instruct the use of water as the solvent of choice. Recent data suggest the role of alternatives to water as a solvent for bowel preparation, with improvements in patient tolerability profiles and on mucosal visualisation.^4^ We therefore performed a systematic review and meta-analysis with the aim of evaluating whether the palatability (taste) and tolerability of bowel preparation may be improved through the use of adjuncts, e.g. flavour enhancers or alternatives to water. In addition, we aimed to assess whether these adjuncts may impact on additional patient-based outcomes, e.g. gastrointestinal adverse events, willingness to repeat bowel preparation, quality of bowel preparation (bowel cleanliness).

## Methods

### Study design

This was a systematic review and meta-analysis of studies reporting on adjuncts which affect the palatability and tolerability of bowel preparation in patients undergoing colonoscopy. We defined adjunct as an agent taken in conjunction with bowel preparation to improve palatability (taste). The systematic review was prospectively registered on The International Prospective Register of Systematic better known as PROSPERO (ID: CRD42020162201) and complies with the Preferred Reporting Items for Systematic Reviews and Meta-Analyses (PRISMA) protocol. Ethical approval was not required for this systematic review.

### Inclusion and exclusion criteria

Study types eligible for inclusion comprised full-text publications of randomised controlled trials, cohort and case-controlled studies. Studies were eligible if data were included on the primary outcome and if they had compared adjuncts ± standard bowel preparation in the intervention arm vs standard bowel preparation (with water) in the control arm. An adjunct was defined as an agent used in conjunction with bowel preparation to improve its palatability. Results were restricted to full-text articles in English.

To restrict the effect studied to adjuncts alone, studies with different dosing regimens or different total volumes of solution in the intervention and control groups were excluded. For example, studies comparing 2 litre (L) polyethylene glycol (PEG) with adjunct vs 4L PEG without the adjunct would be excluded. Studies centred on non-colonoscopic procedures e.g. flexible sigmoidoscopy, Computed Tomography (CT)-colonography, capsule endoscopy or those exclusively enrolling children (<16 years) were also excluded.

### Search strategy

A search strategy was designed based on the Patient, Intervention, Comparator and Outcome (PICO) format. Searches were conducted by two independent researchers (UK and AA) in April 2020 on PubMed, MEDLINE, EMBASE and the Cochrane Database of Systematic Reviews using variations and combinations of the following keywords: bowel preparation, colonoscopy, adjunct, addition, flavour, diluent and solvent (Supplementary Material Figure 1). References within cited papers were also screened for relevant publications using a snowballing approach.

**Figure 1.**
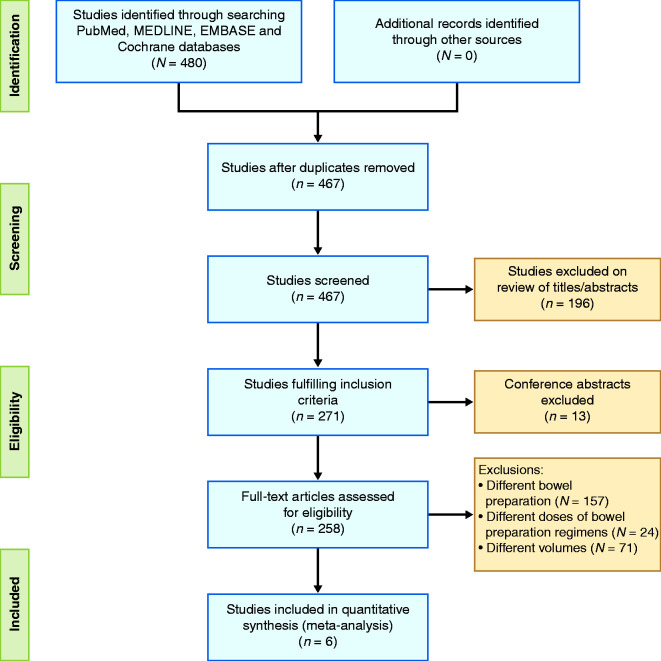
Preferred Reporting Items for Systematic Reviews and Meta-Analyses (PRISMA) flow diagram demonstrating study-selection process based on inclusion and exclusion criteria.

### Outcomes

The primary outcome studied was the palatability of bowel preparation as measured by: (a) patient's perceived rating of bowel preparation, and (b) willingness to repeat bowel preparation in future.

The secondary outcomes included the following: (a) tolerability, i.e. gastrointestinal adverse events (e.g. nausea or vomiting) arising from the bowel preparation, and (b) adequate quality of bowel preparation, as measured using a validated mucosal visualisation scale.

### Data extraction

Data extraction fields included: first author, year of publication, country where study was performed (or of first author), study design, size of the adjunct and control group, description of bowel preparation regimen and volume, outcomes studied and the *n* or summary statistic in intervention vs comparator group for each study outcome.

### Bias assessment

The risk of bias was assessed using the Cochrane risk of bias tool. This was independently assessed by two investigators (IT, AA) and discrepancies were adjudicated via the senior author (KS).

### Data synthesis

Studies meeting the inclusion criteria were analysed using random-effects meta-analysis models. Separate analyses were performed for each of the outcomes being considered. In the case of continuous outcomes, such as those measured on visual analogue scales, where means and standard deviations (SDs) were reported, analyses were performed using random-effects inverse-variance models. Dichotomous variables were presented as odds ratio (OR) with 95% confidence interval (CI) and continuous data reported as mean difference (MD) with 95% CI. Estimates of OR and MD were pooled using a random-effects Mantel-Haenszel model. Heterogeneity was assessed using the *I*^2^-statistic.

Meta-regression models were then produced to estimate the effect of each subgroup of adjunct or bowel preparation separately, and to enable comparisons between these. All analyses were performed using Review Manager 5.3 (Copenhagen: The Nordic Cochrane Centre, The Cochrane Collaboration, 2014).

## Results

### Included studies

In total, the search strategy yielded 467 studies. After exclusions (Figure 1), six studies (*n* = 1187 patients) were included for analysis.^5–10^ All of these were randomised controlled trials (RCTs), conducted between 2012–2016, which analysed the impact of adjuncts on the tolerability and quality of PEG-based bowel preparation. Adjuncts comprised citrus reticulata peel,^6^ orange juice,^7^ menthol candy drops,^8^ simethicone,^5^ Coke Zero^9^ and sugar-free chewing gum.^10^ Only one study^9^ compared different solvents whilst the rest used adjuncts in addition to standard bowel preparation regimens. Study characteristics, including details of the adjuncts, bowel preparation doses and timings are summarised in Table 1.

**Table 1.** Characteristics of studies included in the meta-analysis.

First author, year and country	Study design	Bowel preparation regimen	Adjunct	*n*		Outcomes studied
				Control	Adjunct	
Lan 2012, Taiwan	Single centre, single blinded RCT	PEG/2L, single dose followed by Bisacodyl	Citrus reticulata peel (CRP), 2 g, buccal tablets between drinks	107	105	Effect on tolerability and bowel preparation quality.
Choi 2014, South Korea	Multicentre, single blinded RCT	PEG-ascorbic acid/2L, single dose for morning procedures, split for evening procedures	Orange juice,3 to 4 oz with PEG solution	54	53	Effect on tolerability and patient’s comfort. Quality of bowel preparation: (Aronchick scale).
Sharara 2013, Lebanon	Single centre, single blinded RCT	PEG/4L, split dose	Menthol candy drops, 15 drops to be sucked with PEG solution.	50	49	Palatability and tolerability of bowel preparation (modified Aronchick).
Yoo 2016, South Korea	Single centre, single blinded RCT	PEG-ascorbic acid /2L + 1L additional clear fluid, split dose	Simethicone (2 packs ×200 mg/10 ml added in additional clear fluid)	130	130	Tolerability of bowel preparation, quality of bowel preparation (BBPS).
Seow-En 2016, Singapore	Single centre, single blinded RCT	PEG/2L, single dose	Coke Zero as replacement to water	109	100	Acceptance, tolerability and adverse effects of bowel preparation. Quality as assessed by endoscopist and two endoscopy nurses.
Fang 2016, China	Single centre, single blind RCT	PEG/2L, single dose	Sugar-free chewing gum, 1 piece every 2 h	150	150	Tolerability, quality of bowel preparation (BBPS).

ADR: adenoma detection rate; BBPS: Boston Bowel Preparation Scale; oz: ounce; L: Litre; PEG: polyethylene glycol; RCT: randomised controlled trial.

### Impact on palatability

Palatability (taste) of bowel preparation was measured on a continuous visual analogue scale (VAS) in four studies^5^,^7–9^ and as a categorical outcome (acceptable taste) in two studies.^6^,^10^ One study applied an inverted scale from four to one^9^ which required transformation to enable data synthesis.

#### Palatability

**Figure 2.**
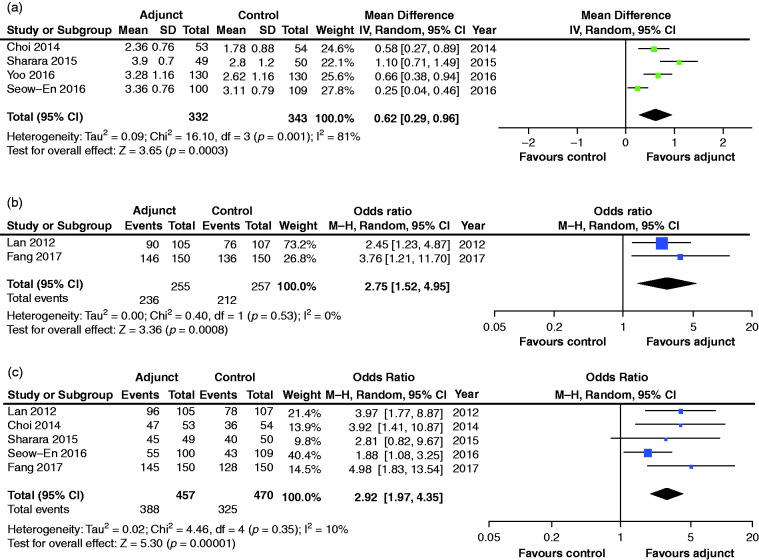
Pooled analyses for palatability score (a), bowel preparation rated as acceptable (b) and willingness to take bowel preparation in future (c). CI: confidence interval; SD: standard deviation; IV: Inverse variance model; MH: Mantel-Haenszel model.

Using an adjusted VAS of one (very low) to five (excellent palatability), pooled palatability scores were significantly higher in the adjunct group, with a mean difference of 0.62 (95% CI: 0.29–0.96, *p* < 0.001) compared to the control arm (Figure 2(a)). The *I*^2^ statistic was 81% indicating considerable heterogeneity.

#### Acceptable taste

The percentage of patients who rated their bowel preparation as having acceptable taste (Figure 2(b)) was significantly higher (OR 2.75, 95% CI: 1.52–4.95, *p* < 0.001) in the adjunct group (92.5%) vs control group (82.5%), with no significant heterogeneity detected (*I*^2^ = 0%).

#### Willingness to repeat bowel preparation

The proportion of patients willing to undergo repeat bowel preparation in future was reported in five studies (Figure 2(c)), of which four ascertained outcomes prior to colonoscopy.^6–8^,^10^ This outcome was significantly higher (OR 2.92, 95% CI: 1.97–4.35, *p* < 0.001) in the adjunct group (84.9%) vs control group (61.9%). No significant heterogeneity was detected in this analysis (*I*^2^ = 10%).

### Impact on tolerability

Five studies (*n* = 887) compared tolerability in terms of nausea, vomiting, bloating and abdominal pain between adjunct and control (standard bowel preparation) groups.^5–9^ All of these side-effects were recorded as categorical variables. One study^10^ was excluded as outcome data were presented as a composite of abdominal pain, bloating and nausea with a corresponding adverse event rate of 41.3% vs 46% (*p* = 0.42) between two groups.

#### Nausea

**Figure 3.**
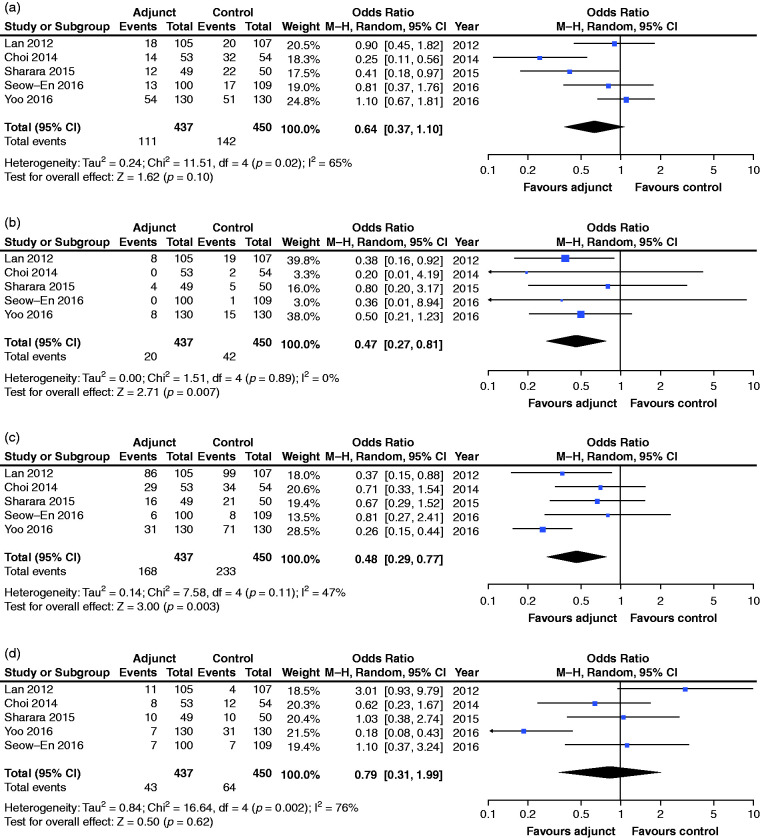
Pooled analyses for tolerability (gastrointestinal adverse events). Nausea (a), vomiting (b), bloating (c) and abdominal pain (d). CI: confidence interval; M-H: Mantel-Haenszel.

Rates of nausea (Figure 3(a)) were not found to differ significantly between the adjunct and control groups (OR 0.64, 95% CI: 0.37–1.10, *p* = 0.10). There was evidence of moderate heterogeneity (*I*^2^ = 65%) in this analysis.

#### Vomiting

Rates of vomiting (Figure 3(b)) were found to be significantly lower in the adjunct group (4.6%) vs control (9.3%) group (OR 0.47, 95% CI: 0.27–0.81, *p* = 0.007). No heterogeneity was identified in this analysis (*I*^2^ = 0%).

#### Bloating

Rates of bloating (Figure 3(c)) were found to be significantly lower in the adjunct group (38.4%) vs control (51.8%) group (OR 0.48, 95% CI: 0.29–0.77, *p* = 0.003). The *I*^2^-statistic was 47% indicating moderate heterogeneity.

#### Abdominal pain

Rates of abdominal pain (Figure 3(d)) were not found to differ significantly between adjunct and control groups (OR 0.79, 95% CI: 0.31–1.99, *p* = 0.62). There was considerable heterogeneity in this analysis (*I*^2^ = 76%).

### Impact on quality of bowel preparation

**Figure 4.**
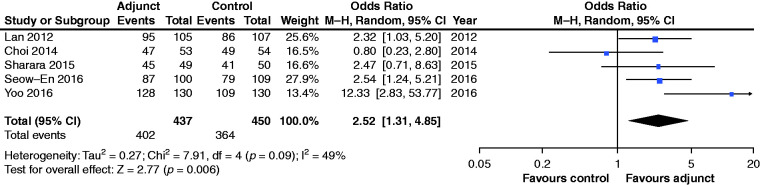
Pooled analyses for adequacy of bowel preparation.CI: confidence interval; M-H: Mantel-Haenszel.

Five studies^5–9^ reported the quality of bowel preparation as the proportion of patients with acceptable or satisfactory bowel preparation, which permitted pooling of this outcome across different studies, despite differences in use of bowel preparation scores between studies. Minor or no bowel staining as assessed by an endoscopist and two nurses,^9^ Boston Bowel Preparation Scale ≥6,^5^ Aronchick scale ≤2,^7^ good or excellent grading on modified Aronchick scale,^8^ grade 1–3 (out of Grade 1–5) for evaluating bowel cleansing^6^ were categorised as adequate bowel preparation. Addition of adjuncts resulted in a higher overall proportion of patients with acceptable bowel cleanliness (92.0% vs 80.9%; OR 2.52; 95% CI: 1.31–4.85, *p* = 0.006) (Figure 4). Heterogeneity was moderate (*I*^2^ = 49%). The study by Fang et al. was excluded from this analysis as it reported the outcome as a continuous variable using the Boston bowel preparation scale. This study found no significant difference (*p* = 0.51) between adjunct and control groups, with median scores of 6.1 and 6.2 respectively.^10^

### Subgroup/sensitivity analyses

As per protocol, subgroup analyses were initially planned to compare different types of adjuncts, i.e. flavour enhancers vs alternative solvents to water. However, only one study had replaced water with another solution, i.e. Coke-Zero.^9^ Sensitivity analysis after excluding this study did not affect the conclusions of results or heterogeneity estimates. Although a meta-regression comparison was intended between studies with an improvement in palatability vs those that did not, no studies were identified for the latter. As such, subgroup comparisons were not performed.

### Quality of evidence

**Figure 5.**
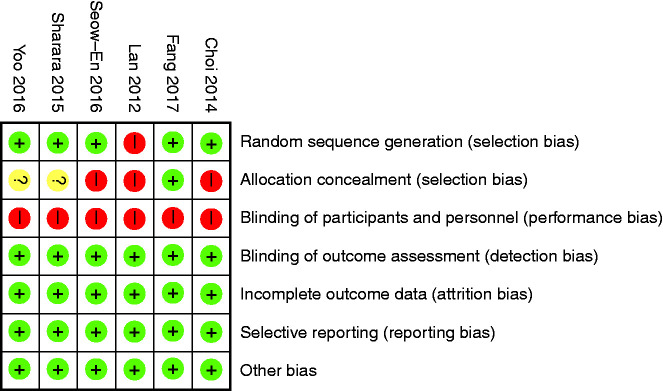
Risk of bias tables.

All included studies were single-blinded RCTs. The risk of bias from most of the included RCTs were low (Figure 5), with the exception of allocation of concealment (selection bias) and blinding of participants due to the nature of studies involving flavour enhancers.

Funnel plots were then produced and analysed for pooled outcome comparisons. There was no evidence of publication bias from the included studies (Supplementary Material Figure 2) for the major outcomes studied.

## Discussion

In this meta-analysis of six RCTs, the use of adjuncts with bowel preparation for colonoscopy was associated with significant improvements in palatability, as measured by the pooled rates of palatability score, acceptable taste and willingness to repeat bowel preparation. Adverse events of vomiting and bloating, but not nausea and abdominal pain, occurred less frequently in the adjunct group. Overall, adequate quality of bowel preparation was more likely to be achieved in the adjunct group compared to controls.

Our findings have direct implications for patients undergoing colonoscopy. First, taste is one of the most burdensome aspects of taking bowel preparation.^4^ In a study by Sharara et al., this was rated by patients as second only to the volume of bowel preparation, with 41.5% assigning a score of 7+ (on a VAS of 0–10 from best to worst) for PEG-based split dose regimens,^4^ which are recommended by the European Society of Gastrointestinal Endoscopy (ESGE)^11^ and used in three of the six included studies. In our analysis, palatability scores and rates of acceptable taste were superior in the adjunct group. Second, patients undergoing colonoscopy may have pre-existing gastrointestinal complaints/complications that can be aggravated by bowel preparation. Vomiting is particularly unpleasant and can arise from noxious stimuli from the gustatory response to bowel preparation.^12^ Importantly, the use of adjuncts reduced the pooled adverse event rates of vomiting (9.3% to 4.6%) and bloating (50.3% to 36.2%). Third, high-quality bowel preparation underpins high-quality colonoscopy.^13^ Adequate bowel preparation was more likely to be achieved in the adjunct group (92.0% vs 80.9%, *p* = 0.006), and probably reflects better tolerability, as poor palatability or vomiting can lead to non-completion of bowel preparation.^14^ In a French survey of 1.12 million colonoscopies, 2% of procedures were repeated due to inadequate bowel preparation.^15^ Improved tolerability may reduce the need for repeat procedures, especially in frailer patients. Finally, the proportion of patients willing to repeat bowel preparation was higher in the adjunct group (84.9% vs 69.1%). This may be particularly beneficial to patients with incomplete examinations or those requiring regular screening or surveillance colonoscopies (e.g. polyp or inflammatory bowel disease surveillance), where long-term patient engagement and compliance is essential.

To our knowledge, only one meta-analysis has been published by Restellini et al.^16^ which evaluated the role of adjuncts as a secondary analysis. However, the authors included studies comparing different regimens and volumes of bowel preparation and therefore could not attribute the reported benefits to adjuncts alone. Our meta-analysis provides novelty as it addresses the issue of confounding by including studies only where bowel preparation regimens and volumes are comparable between intervention and control arms. We also excluded studies which assessed flavour-enhancing adjuncts such as Gatorade,^17^,^18^ olive oil,^19^ pineapple juice^20^ and coffee^21^ due to our exclusion criteria (mainly due to differences in bowel preparation volumes). This was also the case with ascorbic acid, which is a commonly used adjunct. The addition of ascorbic acid to low-volume PEG solution (2L) has been shown to improve taste^22^ and provides similar efficacy in comparison to PEG with 4L solution,^23^,^24^ but these studies did not fulfil eligibility criteria due to differences in PEG volumes, and hence were not included in our meta-analysis. Despite this, pooled effects in favour of patient benefit were demonstrated in most of our studied outcomes, without evidence of publication bias. It is possible that adjuncts do have a role in modulating or counteracting the unfavourable taste profile of conventional bowel preparation.^25^

Our study had several limitations. First, we applied strict selection criteria which led to only six eligible RCTs. There was insufficient data to allow for meaningful subgroup analyses, e.g. by type of adjunct or alternatives to water, or by type of bowel preparation or for additional outcomes which had been specified *a priori*, e.g. adenoma detection rates, hence our deviation from our registered protocol. Second, the heterogeneity of RCTs was variable between outcomes (ranging between 0–80+%) which reflects the differences in the adjuncts studied and reporting of outcomes. This will affect data interpretation. As it is obvious that the adjuncts studied were not the same, the benefit cannot be attributable to any flavour-enhancing adjunct, but regarded as a generalised concept of benefit in carefully selected adjunct methods. We also acknowledge the differences in bowel preparation used between studies (Table 1), which reflects variations in usage worldwide and may contribute to heterogeneity between studies. However, this is negated by our study design, which was intended to study the effect of adjuncts independent of the bowel preparation regimens used. Third, the choice and measurement of outcomes varied across studies. This precluded the ability to pool outcome data across all six studies. Moreover, the willingness to retake bowel preparation may be a composite measure of palatability, tolerability and potentially encompasses the patient experience of colonoscopy, rather than being attributable to palatability alone. Finally, all studies were single-blinded and vulnerable to concealment bias as it was not possible to blind participants to taste.

Many patients and healthcare professionals hold the misconception that bowel preparation should only be used with water. Patient education is key to attaining good quality bowel preparation.^26^ Our meta-analysis shows that adjuncts can be used with bowel preparation in a safe manner without compromising mucosal visualisation but, conversely, increase bowel cleanliness by enhancing palatability and tolerability. This may have implications for the patient perception of colonoscopy and may improve compliance with colonoscopy attendance.

## Conclusion

In this meta-analysis, use of adjuncts with bowel preparation was associated with better palatability, less vomiting and bloating, and superior bowel cleanliness. Adjuncts may be used with bowel preparation to improve the overall patient experience and outcomes of colonoscopy.

## Supplemental Material

sj-pdf-1-ueg-10.1177_2050640620953224 - Supplemental material for Can adjuncts to bowel preparation for colonoscopy improve patient experience and result in superior bowel cleanliness? A systematic review and meta-analysis Click here for additional data file. Supplemental material, sj-pdf-1-ueg-10.1177_2050640620953224 for Can adjuncts to bowel preparation for colonoscopy improve patient experience and result in superior bowel cleanliness? A systematic review and meta-analysis by Umair Kamran, Abdullah Abbasi, Imran Tahir, James Hodson and Keith Siau in United European Gastroenterology Journal
