# Supplementary material for: Can adjuncts to bowel preparation for colonoscopy improve patient experience and result in superior bowel cleanliness? A systematic review and meta-analysis
Source: United European Gastroenterol J. 2020 Aug 24;8(10):1217–27. doi: 10.1177/2050640620953224 (PMC7724533; doi:10.1177/2050640620953224)
Supplement: sj-pdf-1-ueg-10.1177_2050640620953224 - Supplemental material for Can adjuncts to bowel preparation for colonoscopy improve patient experience and result in superior bowel cleanliness? A systematic review and meta-analysis [file sj-pdf-1-ueg-10.1177_2050640620953224.pdf]

SUPPLEMENTARY FILE

(((Bowel prep*).ti,ab OR (colon* prep*).ti,ab)

AND

((adjunct*).ti,ab OR ((flavor*).ti,ab OR (flavour*).ti,ab OR (diluent*).ti,ab OR (solvent*).ti,ab OR (add*).ti,ab)))

AND (colonoscop*).ti,ab"

Supplementary Figure 1: Search strategy

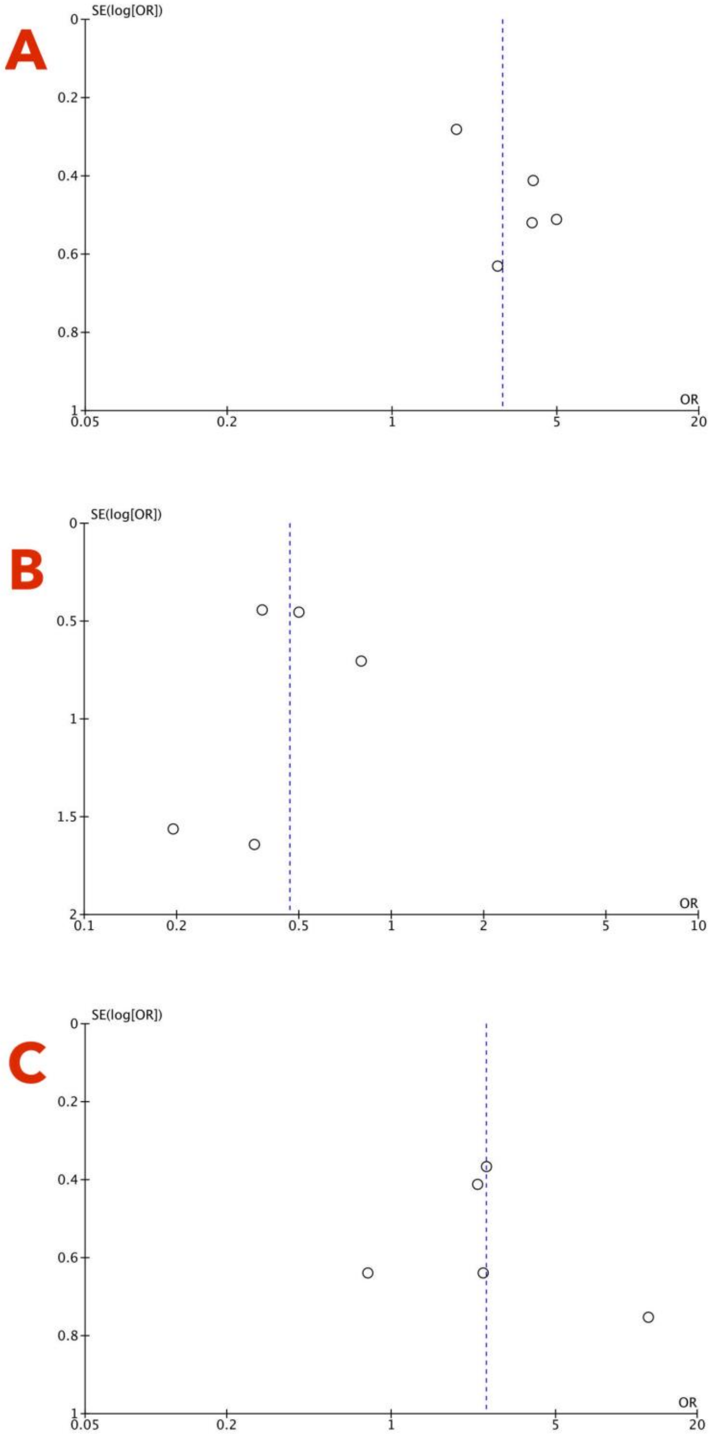

Supplementary Figure 2: Funnel plots of studies evaluating the outcomes of willingness to repeat bowel preparation (**Supplementary Figure 2A**), vomiting (**Supplementary Figure 2B**) and bowel preparation (**Supplementary Figure 2C**).
